# Population-based Surveillance for Hepatitis C Virus, United States, 2006–2007

**DOI:** 10.3201/eid1509.081050

**Published:** 2009-09

**Authors:** R. Monina Klevens, Jeremy Miller, Candace Vonderwahl, Suzanne Speers, Karen Alelis, Kristin Sweet, Elena Rocchio, Tasha Poissant, Tara M. Vogt, Kathleen Gallagher

**Affiliations:** Centers for Disease Control and Prevention, Atlanta, Georgia, USA (R.M. Klevens, J. Miller, T.M. Vogt, K. Gallagher); Colorado Department of Health, Denver, Colorado, USA (C. Vonderwahl); Connecticut Department of Public Health, Hartford, Connecticut, USA (S. Speers); Florida Health Department of Pinellas County, St. Petersburg, Florida, USA (K. Alelis); Minnesota Department of Health, St. Paul, Minnesota, USA (K. Sweet); New York State Department of Health, Albany, New York, USA (E. Rocchio); Oregon Public Health Division, Portland, Oregon, USA (T. Poissant)

**Keywords:** Hepatitis C, epidemiology, surveillance, viruses, USA, dispatch

## Abstract

Surveillance for hepatitis C virus infection in 6 US sites identified 20,285 newly reported cases in 12 months (report rate 69 cases/100,000 population, range 25–108/100,000). Staff reviewed 4 laboratory reports per new case. Local surveillance data can document the effects of disease, support linkage to care, and help prevent secondary transmission.

Hepatitis C virus (HCV) infection is a serious public health problem in the United States and throughout the world. At least 80% of acute infections become chronic (*1*); an estimated 3.2 million persons in the United States alone have chronic HCV infection (*2*). In 2004, an HCV diagnosis was made in 936 of 100,000 outpatient visits for healthcare and in 143 of 100,000 hospital discharges (*3*). This is a chronic infection in which complications are manifested decades after the initial infection. Complications and costs associated with chronic HCV infection are anticipated to increase during 2010–2019 (*4*), because the incidence of new infections peaked from the late 1960s to early 1980s (*5*).

Although identifying persons with HCV infection, including asymptomatic persons, is challenging, the benefits for overall public health make it worthwhile. Infected persons can be referred to care (*6*), treated (if appropriate) (*7*), and counseled to prevent complications. The Centers for Disease Control and Prevention (CDC) and the Council of State and Territorial Epidemiologists recognized these benefits, and in 2003, recommended that past or present infections with HCV (hereafter referred to as HCV infection because most of these cases likely represent chronic rather than acute or resolved HCV infections) become a nationally reportable condition. Surveillance for acute non-A, non-B hepatitis, which was mostly HCV infection, has been performed in the United States since 1982, but in 2007, a total of 33 states also conducted surveillance for HCV infection and reported 133,520 cases to CDC; however, these data remain unpublished.

## The Study

Our study had 2 objectives. The first objective was to describe findings from 6 US state or county health departments that have been funded by CDC to perform enhanced surveillance for HCV infection. The second objective was to discuss the limitations and challenges of conducting population-based surveillance for HCV infection in the United States.

The sites where enhanced hepatitis surveillance was conducted during 2006–2007 were Colorado, Connecticut, Minnesota, New York (excluding New York City), and Oregon; Pinellas County, Florida, a sentinel counties (*8*) site, also contributed hepatitis C reports. The combined population under surveillance from the 5 states and 1 county was an estimated 29.3 million in 2007 (Table). In each of these jurisdictions, clinical laboratories are required to report positive results from HCV assays. For this analysis, a confirmed case of HCV infection was identified in any person who, from July 1, 2006 through June 30, 2007, had at least 1 of the following: 1) a positive result for an HCV recombinant immunoblot assay (RIBA), 2) a positive nucleic acid test (NAT) result for HCV RNA, 3) a documented HCV genotype, or 4) a positive result for a screening test for antibodies against HCV (anti-HCV) with a signal-to-cutoff (s:co) ratio predictive of a true positive result for the given assay.

**Table:** Newly reported cases of past or present HCV infection in 6 US locations, July 1, 2006–June 30, 2007*†

Characteristic	Colorado	Connecticut	Minnesota	New York‡	Oregon	Pinellas County, Florida	Total
Estimated population, 2007	4,862,000	3,502,000	5,198,000	11,068,000	3,747,000	924,000	29,301,000
Year registry initiated	1993	1994	1998	2003	2005	1999	
Sex, no. (%)							
F	1,088 (36.5)	1,360 (35.9)	494 (37.6)	2,882 (30.4)	711 (34.9)	302 (45.4)	6,837 (33.8)
M	1,879 (63.0)	2,429 (64.1)	804 (61.2)	6,595 (69.5)	1,285 (63.0)	359 (54.0)	13,351 (66.0)
Unknown	16 (0.5)	3 (0.1)	15 (1.1)	15 (0.2)	44 (2.2)	4 (0.6)	53 (0.3)
Age group, y, no. (%)							
0–14	16 (0.5)	9 (0.2)	10 (0.8)	14 (0.2)	9 (0.4)	5 (0.8)	63 (0.3)
15–24	126 (4.2)	127 (3.4)	37 (2.8)	318 (3.4)	55 (2.7)	25 (3.8)	688 (3.4)
25–39	638 (21.4)	741 (19.5)	200 (15.2)	1,527 (16.1)	386 (18.9)	69 (10.4)	3,561 (17.6)
40–54	1,645 (55.2)	2,158 (56.9)	798 (60.8)	5,153 (54.3)	1,149 (56.3)	411 (61.8)	11,314 (56.0)
>55	546 (18.3)	755 (19.9)	267 (20.3)	2,438 (25.7)	422 (20.7)	155 (23.3)	4,583 (22.7)
Unknown	12 (0.4)	2 (0.0)	1 (0.1)	42 (0.4)	19 (0.9)	0	76 (0.4)
Case criteria, no. (%)§							
Anti-HCV and supplemental test	593 (19.9)	1,520 (40.1)	520 (39.6)	3,763 (39.6)	203 (10.0)	0	6,599 (32.5)
RIBA	527 (17.7)	466 (12.3)	384 (29.3)	1,404 (14.8)	185 (9.1)	15 (2.3)	2,981 (14.7)
HCV RNA	1,245 (41.7)	1,984 (52.3)	928 (70.7)	5,831 (61.4)	818 (40.1)	28 (4.2)	10,834 (53.4)
Genotype	586 (19.6)	21 (0.6)	304 (23.2)	1,473 (15.5)	207 (10.2)	142 (21.4)	2,733 (13.5)
Anti-HCV and s:co ratio	1,859 (62.3)	1,352 (35.7)	253 (19.3)	4,709 (49.6)	905 (44.4)	521 (78.4)	9,599 (47.3)
Source of report, no. (%)							
Laboratory	2,561 (85.9)	3,792 (100.0)	878 (66.9)	8,252 (86.9)	1,923 (94.3)	592 (89.0)	17,998 (88.7)
Others, combined¶	422 (14.2)	0	435 (33.1)	1,240 (13.1)	117 (5.7)	73 (11.0)	2,287 (11.3)
Total reports	2,983	3,792	1,313	9,492	2,040	665	20,285
Report rate/100,000 population	61.4	108.3	25.3	85.8	54.4	71.9	69.2

*HCV, hepatitis C virus; RIBA, recombinant immunoblot assay; anti-HCV, antibodies against HCV; s:co, signal-to-cutoff. †A confirmed case requires laboratory confirmation. Laboratory criteria consist of at least 1 of the following: a positive result for a HCV RIBA, a positive result for a nucleic acid test for HCV RNA, an HCV genotype, or enzyme immunoassay with detection of anti-HCV and a s:co ratio predictive of a true positive result for a particular assay (see www.cdc.gov/ncphi/disss/nndss/casedef/hepatitisccurrent.htm for the 2005 Council of State and Territorial Epidemiologists/Centers for Disease Control and Prevention case definition). ‡Excludes New York, New York. §Cases could be reported with more than minimum laboratory criteria; totals add up to >100%. ¶Other sources of reports included private healthcare providers, facilities such as hospitals and outpatient clinics, and institutions such as prisons, blood banks, and drug treatment centers, among others.

Laboratories and providers continuously reported positive results for HCV markers (e.g., anti-HCV, RIBA, NAT, genotype) to state or local health departments. Health department staff checked patients’ names and dates of birth from each report against a surveillance database to determine whether a case had been previously reported. Newly reported cases (i.e., previously not captured in the database of this jurisdiction) were entered into this database along with hepatitis test results. Health department staff investigated cases and collected basic demographic and clinical information to confirm the case definition and to epidemiologically describe the case. We calculated rates of newly reported cases by using denominators available from the 2007 population estimates from the US Bureau of the Census (www.census.gov/compendia/statab).

Two supplemental assessments were conducted. The first assessment measured the number of laboratory reports associated with each new case. Staff at each site monitored a convenience sample of laboratory reports and measured the number excluded, reasons for exclusion, and the number that eventually were classified as newly reported cases. The second assessment determined the validity of basic epidemiologic information. For this task, CDC drew a random sample of 10 cases per site from among those reported during the 12-month reporting period (n = 60) and extracted the following variables: date of birth, county of residence, sex, race, and clinical test results associated with HCV infection. Surveillance staff contacted at least 1 healthcare provider to independently collect this information. We measured agreement between the information initially reported and the information collected during the validation using a κ statistic (*9*).

The 6 sites reported a total of 20,285 cases of confirmed HCV infection that were previously unreported in their respective jurisdictions (Table). Of these, 66% of case-patients were male and 56% were 40–54 years of age (men and women combined) (Table). More than half (52%) of the reports lacked information on race or ethnicity. Most cases (89%) were reported by clinical laboratories. The laboratory criterion most frequently reported was a positive result for HCV RNA (53%). The rate of new reports of past or present HCV infection was 69/100,000 population (range 25–108/100,000).

Sites monitored all incoming reports on average for 8 days (range 5–16 days). A total of 2,180 reports were received and, among these, 491 (23%, range 13%–52%) met the case definition and were considered newly reported cases; Oregon had the highest proportion of newly reported cases (52%) and the newest registry. The remaining reports fell into the following categories: already in the database (68%, range 30%–78%), lacking value for s:co ratio (5%, range 3%–13%), negative test results for an HCV marker (2%, range 1%–4%), or missing key demographic data (1%, range 0%–2%).

All cases were confirmed to meet the case definition. Agreement was high for age (κ = 1.0, p<0.001), sex (κ = 0.96; p<0.001), and county of residence (κ = 1.0; p<0.001); county data were missing for 6 (10%) cases.

## Conclusions

We documented that for every 4 laboratory reports, ≈1 newly reported case of HCV infection was identified. The overall annual rate of new case reports was 69/100,000 population in 6 sites that were conducting enhanced surveillance. In the 4 states (Colorado, Connecticut, Minnesota, Oregon) for which comparable data were available, the number of newly reported cases of HCV infection was at least 4× the number of newly reported HIV infections in 2006 (*10*). The 1 county in Florida was not included in the comparison because no HIV data were available.

Two limitations must be mentioned. First, we do not know how many of the newly reported cases represent current infections. In the United States, 80% of prevalent anti-HCV–positive cases are HCV RNA positive (*2*); thus, most laboratory confirmed cases reported to surveillance are likely chronic infections, but could also represent acute or resolved infections. Electronic laboratory reporting is the most efficient way to identify potential cases (*11*), but because no current laboratory test can distinguish acute from chronic HCV infections, identification of acute-phase cases requires contacting the provider or patient to determine whether acute symptoms were present. Due to the high volume of reports received, this level of follow-up was not routinely conducted.

The second major limitation is that testing patterns in the community are unknown. Providers are inconsistent about eliciting risk factor information and about testing and referring patients to specialists (*12*). Patient access to care and structural factors in institutions (e.g., incentives and disincentives for testing at jails, prisons, and drug treatment programs) and in the community (e.g., screenings) also affect testing and, therefore, the reporting rate.

The greatest value of conducting surveillance for chronic HCV at the state and local level is to measure local frequency of disease. Local and state health departments share information such that changes of residence of cases within the state over time would not result in a duplicate case count. However, in aggregating these data at the national level, an infected person who moved from 1 state to another would likely trigger a new report in another state, thus resulting in an overestimate of the national prevalence. Therefore, as a coordinated surveillance system for chronic HCV is developed, a mechanism to prevent duplication of cases across states will need to be developed.

Many factors affect case reporting, such as, local public health reporting requirements, the sophistication and capacity of laboratories to electronically report de-duplicated positive test results, availability of health department staff to conduct investigations and follow-up on reports, time since registry was initiated, and the capacity of the system to maintain ongoing surveillance efforts. Without an understanding of these factors, interpreting the meaning of new HCV infection case reports is difficult.

Local health departments need chronic HCV infection surveillance to document effects of disease, identify persons in need of linkage to care, and prevent complications among persons infected (*13*). However, accurately collecting the necessary information is challenging for health departments, and we currently lack evidence that obtaining these data will result in a lower incidence of illness and death. A full assessment of the benefits and costs of conducting comprehensive surveillance for chronic HCV infection is overdue. Currently, the enhanced hepatitis surveillance sites are developing recommendations for best practices and plan to share methods and tools with all interested health departments. Future studies should evaluate what level of surveillance for chronic HCV is feasible and whether the prevention benefit is worth the effort.
